# The spectrum of neurological derangement due to fat embolism: a case series

**DOI:** 10.3389/fneur.2026.1752690

**Published:** 2026-07-28

**Authors:** Fatma Shalabi, Noor Dirini, Mohamad Assayuri, Moshe Gomori, Peter Vernon van Heerden

**Affiliations:** 1 Department of Neurology, Hadassah-Hebrew University Medical Center, Jerusalem, Israel; 2 Department of General Intensive Care, Hadassah-Hebrew University Medical Center, Jerusalem, Israel; 3 Department of Anesthesiology, Critical Care, and Pain Medicine, Hadassah-Hebrew University Medical Center, Jerusalem, Israel; 4 Department of Neuroradiology, Hadassah-Hebrew University Medical Center, Jerusalem, Israel

**Keywords:** cerebral fat embolism, fat embolism syndrome, imaging features, long bone fractures, neurological manifestation, rapid onset coma, trauma

## Abstract

Cerebral fat embolism (CFE) is a rare neurological feature of fat embolism syndrome (FES), often occurring after long bone fractures. It has a mortality rate of approximately 10% and presents with a spectrum of symptoms, ranging from mild confusion to sudden unresponsiveness and death. Diagnosis primarily relies on clinical history and findings, especially when magnetic resonance imaging is unavailable. We report three unique cases involving traumatic long bone fractures in which patients showed varying severity of CFE, including rapid loss of consciousness. These cases highlight the diverse presentations and outcomes of CFE, including associated symptoms, imaging features, treatment approaches, and prognosis.

## Introduction

Fat embolism syndrome (FES) occurs in both non-traumatic and traumatic patients, with a notably higher incidence among individuals with pelvis and long bone fractures, particularly involving the femur (1). Additional risk factors include young male patients, typically with a median age of 39 years (1), hypovolemia, and systemic stress responses (2).

FES may develop in up to 85% of trauma patients within 48 h following injury (3), most frequently presenting as respiratory derangement. Other common signs include neurological deficits, cardiovascular instability, skin petechiae, conjunctival signs, and fever (1, 2, 4). Two primary theories explain the underlying pathophysiology of FES. The mechanical theory suggests that fat droplets are directly released from fractured bones into the venous system, leading to mechanical blockage of capillaries and smaller blood vessels in various organs. Conversely, the biochemical theory proposes that a profound inflammatory response occurs due to free fatty acids entering the bloodstream, increasing capillary permeability and causing tissue damage, primarily in the lungs but also in other organs (5–7).

The diagnosis of FES is primarily clinical, with diagnostic criteria proposed by Gurd and Wilson (8), Schonfeld (9), and Lindeque et al. (10) (Table 1). Among these, the Gurd and Wilson criteria are the most widely adopted; however, none have been universally validated. The modified Gurd’s criteria (Table 1) emphasize more extensive neurological assessment and neuroimaging findings (11).

**Table 1:** Gurd and Wilson’s (8), modified Gurd’s (11), and Schonfeld’s (9) criteria.

Gurd and Wilson’s	Modified Gurd’s	Schonfeld’s
*Major criteria*	*Major criteria*	*Major criteria*
Hypoxia (PaO_2_ < 60 mmHg; FiO_2_ = 0.4)	Hypoxia (PaO_2_ < 60 mmHg; FiO_2_ = 0.2)	Hypoxia ((PaO_2_ < 70 mmHg)
Cerebral symptoms unrelated to head injury	Altered mentation with compatible changes in MRI	Diffuse infiltrate on chest X-Ray
Petechial rash	Petechial rash on conjunctiva and upper trunk	Petechial rash
*Minor criteria*	*Minor criteria*	*Minor criteria*
Tachycardia (>110 bpm)	Tachycardia (>100 bpm)	Tachycardia (>120 bpm)
Pyrexia (>38.5 °C)	Pyrexia (>38 °C)	Pyrexia (>38 °C)
Jaundice	Renal changes	Tachypnea
Renal changes	Retinal embolism	Mental confusion
Retinal embolism	Unexplained anemia	
Sudden unexplained anemia	Thrombocytopenia	
Sudden thrombocytopenia		
Elevated ESR (>71 mm/h)		
Fat macroglobulinemia		

PaO_2_, partial pressure of oxygen; FiO_2_, fraction of inspired oxygen; bpm, beats per minute; ESR, erythrocyte sedimentation rate; MRI, magnetic resonance imaging.

## Cerebral fat embolism (CFE)

Isolated neurological manifestations have been documented in trauma patients with long bone fractures, with incidence rates of approximately 0.9–2.2% (12). CFE often manifests within 24 h after admission (12) and may present either as focal or generalized neurological deficit. Focal signs, such as hemiparesis, speech disturbances, or seizures, may follow ischemic or hemorrhagic stroke (13). In contrast, generalized symptoms such as headache, confusion, hallucinations, agitation, and rapid alteration of consciousness (even coma) may follow diffuse brain injury (4, 12, 14). Although fatal cases are rare, they have been reported (7, 15). Additional uncommon neurological symptoms include paroxysmal sympathetic hyperactivity syndrome (16), dystonia, and decorticate posturing (12).

Imaging studies reveal that brain CT scans generally have limited diagnostic utility; they often show normal findings, despite neurological symptoms. When abnormalities are present, they may present non-specific edema or more characteristic patterns such as hypodense, round lesions with fat density/attenuation or hypodense middle cerebral artery (MCA) signs suggestive of macroscopic emboli containing fat (12). Magnetic resonance imaging (MRI) remains the most sensitive and gold-standard imaging modality in diagnosing CFE. It has characteristic features such as a starfield-like pattern caused by multiple small hyperintense restricted diffusion lesions on diffusion-weighted imaging (DWI) and hypointense lesions on susceptibility-weighted imaging (SWI). These lesions can be located in cortical and subcortical regions across different vascular territories, appearing early in CFE (4, 12). Petechial hemorrhages can be seen on SWI. Subacute stages may demonstrate confluent, symmetric cytotoxic edema and vasogenic edema, with hyperintense signals on T2-weighted imaging. Chronic stages might show signs of demyelination (12). Transcranial Doppler ultrasound (TCD) may detect microembolic signals and patent foramen ovale (PFO), while electroencephalography (EEG) may be useful for excluding seizures (12).

The mortality rate from CFE in orthopedic patients is 10% (12), but in non-orthopedic cases, especially among the elderly with comorbidities, it may reach approximately 30% (1).

## Case presentations

### Case 1

A young, previously healthy patient sustained multiple injuries following an explosion. The patient was fully awake when initially transferred to another facility. An urgent exploratory laparotomy was performed following a positive FAST ultrasound examination, which revealed no intra-abdominal injury. Postoperation whole-body CT identified a right sacral fracture, arterial injuries to both lower extremities, and complex comminuted fractures of the right femur and tibia/fibula, without pathological findings in the head. External fixation of the leg fractures and deep posterior compartment fasciotomy were then urgently performed. The patient was admitted to our intensive care unit (ICU) on POD 2, sedated, mechanically ventilated, and hemodynamically stable. Subsequent internal fixation of the tibia on POD 4, left lower extremity vascular bypass on POD 10, and additional wound care procedures were undertaken.

**Figure 1:**
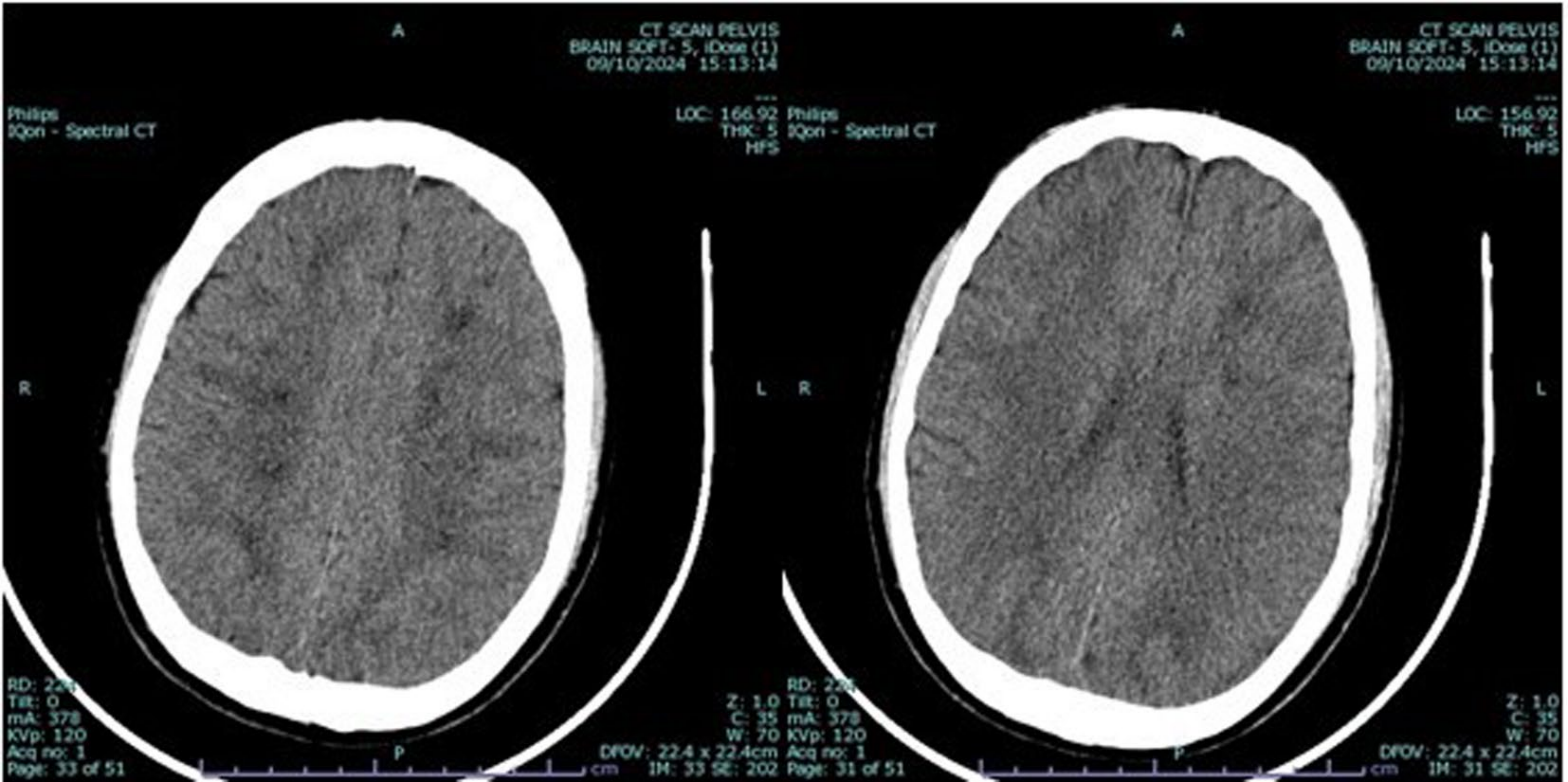
Different levels of axial brain computed tomography (CT) of Case 1 shows bilateral and symmetric multifocal hypodensities in the frontal and parietal subcortical (arrow) and deep white (arrow) predominantly in the centrum semiovale and involving the bilateral caudate heads, internal capsules, left thalamus, and cerebellum.

Upon attempts to wean sedation, the patient remained unresponsive. A head CT showed bilateral, symmetric, multifocal hypodensities mainly in white matter regions, involving the centrum semiovale, caudate heads, internal capsules, thalamus, and cerebellum (Figure 1). These imaging findings raised the suspicion of CFE, promoting further evaluation for FES. Ophthalmologic exam was normal; cutaneous signs were absent. EEG lacked epileptiform activity. During the ICU stay, the patient developed ventilator-associated pneumonia, with new consolidations on chest radiograph. Tracheostomy was subsequently performed. Over 40 days of ICU admission, the patient showed gradual neurological improvement, regaining consciousness, obeying commands, and speaking short but coherent sentences before being discharged to the Orthopedic Department and later on for rehabilitation.

### Case 2

**Figure 2:**
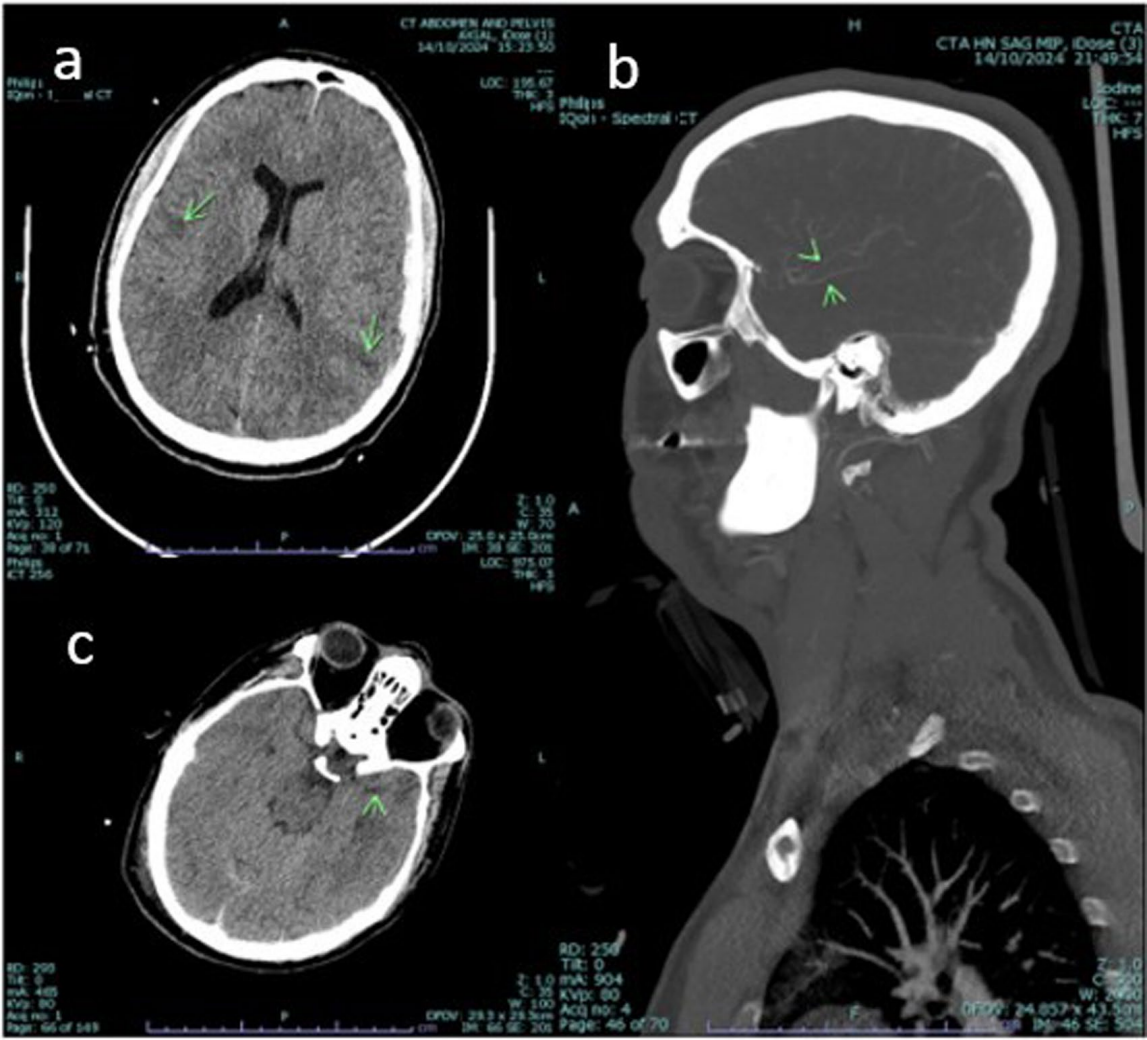
Brain CT of Case 2. **(a)** Axial slides show scattered subcortical hypodense areas in the bilateral cerebral hemispheres; **(b)** Sagittal CTA shows narrowing and irregularities of the MCA branches; **(c)** Axial slides show a linear hypodensity at the left Sylvian fissure suggestive of hypodense MCA sign. CT, computed tomography; CTA, CT angiography; MCA, middle cerebral artery.

A young, previously healthy patient was involved in a high-velocity motor vehicle accident (MVA). Upon arrival at the Emergency Department (ED), the patient exhibited altered mental status, with a Glasgow Coma Scale (GCS) of 13. The patient rapidly became hemodynamically unstable, necessitating emergent intubation, ventilation, and resuscitation. A whole-body CT scan revealed a left-sided subdural hematoma with midline shift, along with scattered subcortical hypodense areas in both cerebral hemispheres (Figure 2a). Additional findings included a small left-sided pneumothorax, left posterior rib fractures (ribs 8–11), bilateral pulmonary contusions, bilateral mid-shaft femoral fractures, and a right tibia/fibula fractures (Gustilo grade III). Head and neck CT angiography (CTA) showed no vascular injury. The patient underwent urgent surgery for placement of a right external ventricular drain, external fixation of the right tibia and fibula, and bilateral femoral fixation. Postoperative head CT confirmed effective decompression of the subdural hematoma without parenchymal damage, and a follow-up imaging showed resolution of the hematoma. However, new small hypodense areas appeared in the anterior temporal lobes, slightly larger on the left side. Therefore, a CTA was performed and was reported as normal, with no evidence of large-vessel occlusion. The patient was successfully weaned from sedation and extubated on day 10.

On day 11, a neurological consultation was obtained because of suspected right arm weakness. Neurological examination revealed a National Institutes of Health Stroke Scale (NIHSS) score of 19, with symptoms including global aphasia, mild right facial palsy, right arm weakness, and paraplegia (attributed to orthopedic injuries). Head CT demonstrated extensive hypodensity in the left temporal and parietal lobes. Follow-up CTA and digital subtraction angiography excluded large-vessel occlusion or dissection, while TCD excluded vasospasm and PFO. Cardiac evaluations, including transthoracic echocardiogram and continuous electrocardiography monitoring, showed no embolic source or arrhythmias. Ophthalmologic and dermatologic evaluations were unremarkable. Given the lack of other etiologies, CFE was considered as the most likely explanation for the neurological deterioration. A review of the brain imaging showed that the initial CTA demonstrated narrowing and irregularities in the MCA branches (Figure 2b). In addition, the non-contrast CT performed 2 days after admission showed a linear hypodensity at the left Sylvian fissure, suspicious for a hypodense MCA sign (Figure 2c), a rare radiological sign of fat embolism (13).

The patient developed ventilator-associated pneumonia during the ICU stay. On day 20, the patient underwent internal fixation of his lower limb fractures. Prior to transfer to the orthopedic ward, neurological status gradually improved, with partial recovery of speech and the right-sided motor function. Later on, the patient was transferred to rehabilitation.

### Case 3

A young, previously healthy patient was involved in a high-energy MVA. Upon arrival at the ED, the patient had a GCS score of 10, was breathing spontaneously, and had normal oxygen saturation. A FAST examination was positive, and pelvic X-ray revealed an open-book fracture. Whole-body CT showed a small left-sided pneumothorax, bilateral pulmonary contusions, a grade IV splenic laceration, grade I hepatic laceration, free intra-abdominal fluid, an open-book pelvic fracture, bilateral femoral shaft fractures, and a right tibia/fibula fracture. Head CT scan excluded acute hemorrhage. A pelvic binder was applied, and the patient was rushed to surgery, where an exploratory laparotomy identified a retroperitoneal hematoma without active bleeding. Splenectomy was performed, followed by abdominal closure. External fixation was applied to the bilateral femoral fractures and the right tibia/fibula.

Postoperatively, the patient was admitted to the ICU, intubated, and hemodynamically stable on minimal norepinephrine support following transfusions. On POD 1, the patient self-extubated and was alert, oriented, and hemodynamically stable, with no respiratory distress. The patient received intermittent morphine for pain relief, with no respiratory signs. Approximately 6 h later, the patient became suddenly unresponsive, with GCS score dropping to 7. The pupils were bilaterally reactive but midsized, and blood gasses, vital signs, and chest X-ray remained normal. Naloxone had no effect, and the patient was re-intubated.

**Figure 3:**
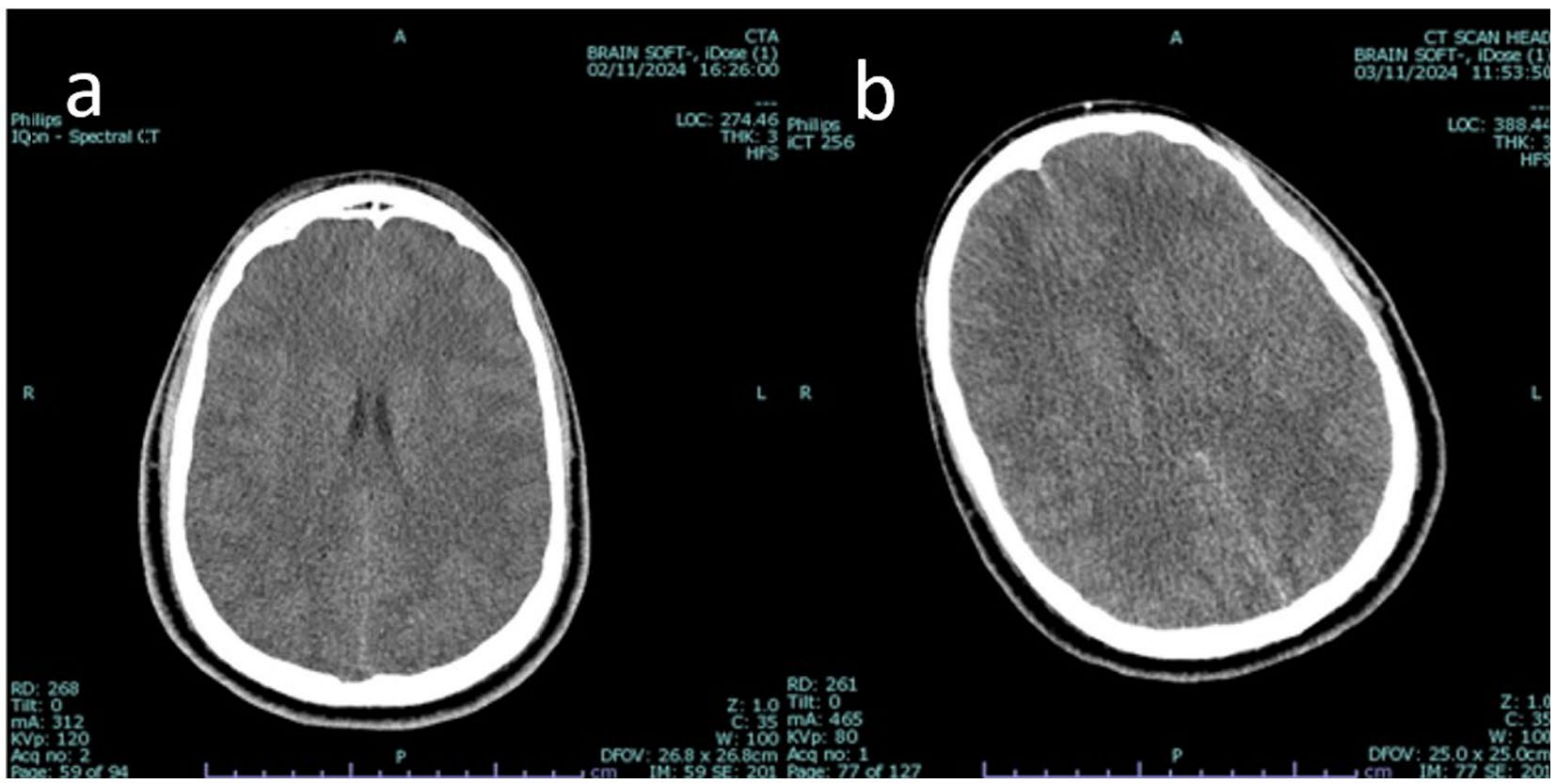
Two slides of axial brain computed tomography of Case 3. **(a)** Diffuse cerebral edema with effacement of the ventricles. **(b)** Worsening of the diffuse cerebral edema with complete effacement of the ventricles.

Urgent comprehensive imaging, including whole-body CT with pulmonary embolus protocol and CTA, was performed and was unremarkable. Head CT revealed diffuse cerebral edema with ventricular effacement (Figure 3a). A “wake-up test” afterwards showed a GCS score of 3 with loss of brainstem reflexes. Later, neurological examination demonstrated bilaterally non-reactive dilated pupils. A repeated head CT revealed worsening cerebral edema with complete ventricular effacement (Figure 3b), resulting in brainstem compression. EEG showed no epileptiform discharges or evidence of status epilepticus. A petechial rash on the anterior chest wall supported the diagnosis of FES. Despite corticosteroid therapy, the patient’s neurological status did not improve. The patient developed diabetes insipidus, which was managed with low-dose desmopressin. On day 20, orthopedic internal fixation was undertaken. The patient was ultimately transferred for palliative care and subsequently died.

## Discussion

The prevalence of FES, including CFE, is higher in patients with pelvis and long bone fractures, and it typically manifests early, usually within the first 48 h. All of our patients had severe long bone fractures, and in two of them, neurological symptoms appeared early during admission. The second patient developed CFE later in the hospital course, but delayed onset has been previously described in the literature (4). Early external fixation was performed to stabilize fractures; however, it did not prevent FES in our cases, likely because of the significant inflammatory response and trauma severity, which may promote dispersion of fat droplets into the bloodstream.

Respiratory involvement is the most common manifestation of FES. In the first two cases, chest CT demonstrated pulmonary abnormalities without significant respiratory compromise. The third patient showed no pulmonary involvement. Clinically, confusion and the absence of focal neurological signs are more specific for CFE, although stroke has also been reported (13). The second patient exhibited focal signs consistent with an MCA infarct, confirmed by head CT and hypodense MCA sign in CTA. Although coma is rarely the initial presentation of CFE, it may occur, especially in patients with other risk factors, due to cerebral edema and/or encephalitis secondary to the cytotoxic effects of free fatty acids (2, 14, 17). The rapid loss of consciousness in the third case, without cardiac or respiratory cause, raised suspicion of CFE. Diffuse axonal injury (DAI) is a differential diagnosis in trauma patients, but the intact neurological presentation of the first patient and the rapid follow-up neurological improvement in the second and third cases, prior to sudden deterioration, suggest that DAI was unlikely.

Diagnosis was based on the patient history and clinical presentation, supported by imaging, mainly brain MRI, and by exclusion of other causes. Although CFE is rarer than FES, neurological deterioration and its severe manifestations predominated in our cases, as previously documented (13, 14, 17). Due to technical limitations, we could not perform brain MRI but relied on clinical findings, timing, and initial CT scans to support the diagnosis of CFE (5), in addition to ruling out other suspected diagnoses, such as DAI in cases 1 and 3 and thromboembolic stroke in case 2.

Treatment remains mainly supportive, focusing on adequate oxygenation, ventilation, and sedation (12). Steroids (1, 18), heparin (19), and statins remain controversial (12). We administered corticosteroids in the third case as a trial because of the severe neurological manifestation, but no improvement was observed, likely due to irreversible ischemic damage from diffuse cerebral edema. Early fracture fixation within 24 h is typically recommended to reduce complications, particularly pulmonary complications (12, 20); however, it did not prevent severe neurological outcomes in our cases.

Although CFE can be fatal, it is usually self-limiting, especially with supportive care and rehabilitation. Two of our patients had good recoveries and were discharged to rehabilitation. The non-survivor patient survived only through the ICU and acute phase.

## Conclusion

CFE is a rare complication of FES with variable clinical presentations and a high mortality rate. Therefore, awareness of this syndrome and recognition of its risk factors are important for reducing the severity of CFE. Brain CT and CTA may help exclude competing diagnoses in cases of acute neurological deterioration, but normal CT does not exclude cerebral fat embolism; MRI remains the preferred diagnostic modality when feasible. The findings of early signs of CFE prompt closer monitoring and early neurological consultation. Further studies on the prevention and treatment of FES are needed to improve outcomes and prevent this potentially fatal condition.

## Data Availability

The original contributions presented in the study are included in the article/supplementary material, further inquiries can be directed to the corresponding author.
